# Measuring the global, regional, and national burden of multiple myeloma from 1990 to 2019

**DOI:** 10.1186/s12885-021-08280-y

**Published:** 2021-05-25

**Authors:** Linghui Zhou, Qin Yu, Guoqing Wei, Linqin Wang, Yue Huang, Kejia Hu, Yongxian Hu, He Huang

**Affiliations:** 1 Bone Marrow Transplantation Center, The First Affiliated Hospital, School of Medicine, Zhejiang University, No.79 Qingchun Road, Hangzhou, China; 2 Institute of Hematology, Zhejiang University, Hangzhou, China; 3 Zhejiang Province Engineering Laboratory for Stem Cell and Immunity Therapy, Hangzhou, China; 4 Zhejiang Laboratory for Systems & Precision Medicine, Zhejiang University Medical Center, Hangzhou, China; 5 College of Life Science, Zhejiang Chinese Medical University, Hangzhou, China

**Keywords:** Global burden of disease, Multiple myeloma, Incidence, Death, Disability adjusted life-years

## Abstract

**Background:**

Multiple myeloma (MM) is a major health concern. Understanding the different burden and tendency of MM in different regions is crucial for formulating specific local strategies. Therefore, we evaluated the epidemiologic patterns and explored the risk factors for MM death.

**Methods:**

Data on MM were collected from the 2019 Global Burden of Disease study. We used incidence, mortality, and disability adjusted life-years to estimate the global, regional, and national burden of MM.

**Results:**

In 2019, there were 155,688 (95% UI, 136,585 – 172,577) MM cases worldwide, of which 84,516 (54.3%, 70,924 – 94,910) were of men. The age-standardized incidence rate (ASIR) was 1.72/100,000 persons (95% UI, 1.59–1.93) in 1990 and 1.92/100,000 persons (95% UI, 1.68–2.12) in 2019. The number of MM deaths increased 1.19-fold from 51,862 (95% UI, 47,710–58,979) in 1990 to 113,474 (95% UI, 99,527 – 121,735) in 2019; the age-standardized death rate (ASDR) was 1.42/100,000 persons (95% UI, 1.24–1.52) in 2019. In recent 15 years, ASDR showed a steady tendency for men, and a downward tendency for women. Countries with high social-demographic indexes exhibited a higher ASIR and ASDR. Australasia, North America, and Western Europe had the highest ASIR and ASDR, with 46.3% incident cases and 41.8% death cases. Monaco had the highest ASIR and ASDR, which was almost half as high as the second highest country Barbados. In addition, United Arab Emirates and Qatar had the largest growth multiple in ASIR and ASDR, which was twice the third country Djibouti.

**Conclusions:**

Globally, incident and death MM cases have more than doubled over the past 30 years. The increasing global burden may continue with population aging, whereas mortality may continue to decrease with the progression of medical technology. The global burden pattern of MM was diverse, therefore specific local strategies based on different burden patterns for MM are necessary.

**Supplementary Information:**

The online version contains supplementary material available at 10.1186/s12885-021-08280-y.

## Background

Multiple myeloma (MM) is one of the most frequent hematological malignancies worldwide, ranking 24th among the most common cancers. In 2018, the number of new cases attributed to MM (159,985 new cases, accounting for 0.9% of all new tumors) was almost 1.5 times that of deaths (106,105 new cases account for 1.1% of all cancer deaths) [1]. The incidence rate of MM is the highest in North America, Australia, New Zealand, and Europe, whereas it is the lowest in Asia (except for Western Asia) [2]. The median age at diagnosis of MM is approximately 70 years; 37, 26, and 37% of MM patients were < 65, 65–74 years, ≥ 75 years, respectively [3]. MM is extremely rare in patients aged < 30 years, with an incidence of 0.02–0.3%, which is somewhat higher in males than in females [4]. In recent years, with the progress in autologous hematopoietic stem cell transplantation (auto-HSCT) and new agents, the overall survival of myeloma has significantly prolonged [5–7]. According to the Surveillance, Epidemiology, and End Results (SEER) data, the 5-year survival rate of MM was 25% in 1975–1977 and 27% in 1987–1989, increasing to 49% during 2005–2011 [8]. Interestingly, bortezomib and thalidomide/lenalidomide were approved in 2003 and 2006 for the treatment of MM, respectively. These conclusions refers to different periods of time, so current global burden trend should be considered.

For understanding the epidemiologic patterns, trends and risk factors of multiple myeloma across various sex, age groups, social-demographic index and location over the past 30 decades. We conducted the study to evaluate the epidemiologic patterns and explore the risk factors attributed to MM death.

## Methods

### Study data

We obtained information on the annual incidence, mortality, and disability adjusted life-years (DALY) of MM between 1990 and 2019 from the Global Health Data Exchange (GHDx) query tool (http://ghdx.healthdata.org/gbd-results-tool). Information on sex, age, and risk factors were also collected to assess the burden of MM. The study was based on an analysis of estimates from the GBD study 2019. To further analyze the global burden of MM, we classified disease information according to the following three criteria. First, we mapped the world to assess the incidence and mortality of MM in 204 countries and territories as well as the corresponding percentage change over the past 30 years. Second, we divided 204 countries and territories into five categories, according to the socio-demographic index (SDI), the geometric average of total fertility, per capita income, and average years of education, ranging from zero to one. The larger the SDI, the more developed the country [9, 10]. According to the data source, the world was divided geographically into 21 regions to assess the differences.

### Statistical analysis

Age-standardization refers to the method of statistical processing of demographic data according to the same standard age composition. The purpose is to eliminate the influence of different age composition of the population and ensure the comparability of statistical indicators. The age-standardized incidence/death/DALY rates (ASRs) (per 100,000 population) equals the sum of the events of the age group i ratio (a_i_) and the number (w_i_) of the standard population group i divided by the sum of the number of the standard population, that is, $\mathrm{ASR}=\frac{\sum_{i=1}^A{a}_i{w}_i}{\sum_{i=1}^A{w}_i}\times \mathrm{100,000}$. Uncertainty interval reflects the certainty of an estimate, which is calculated 1000 times, each time sampling from distributions rather than point estimates for data inputs, data transformations and model choice. DALYs were equal to the sum of the years lived with disability and the years of life lost [9]. Risk factor was defined as an attribute, behavior, exposure, or other factor which is causally associated with an increased (or decreased) probability of a disease or injury. According to the GBD definition standard, high body-mass index was defined as BMI > = 25. Based on these data, we visualized and compared ASRs across different age, sex, SDI, 21 regions and countries to find differences and change trends for formulation of specific local strategies. The death and DALYs attributable to risk factor for MM across different sex and regions were also compared. Furthermore, we used the joinpoint regression model (version 4.7.0.0) to calculate the trends in incidence and mortality. In addition, we plotted scatter plots to assess the association between percentage change from 1990 to 2019 and ASR and SDI, respectively. The SDI in 2019 was used as an indicator of the health care level of each country. ρ refers to Person’s correlation coefficient. All calculations were performed using R software (version 3.5.1) [11].

## Results

### Incidence burden

**Fig. 1:**
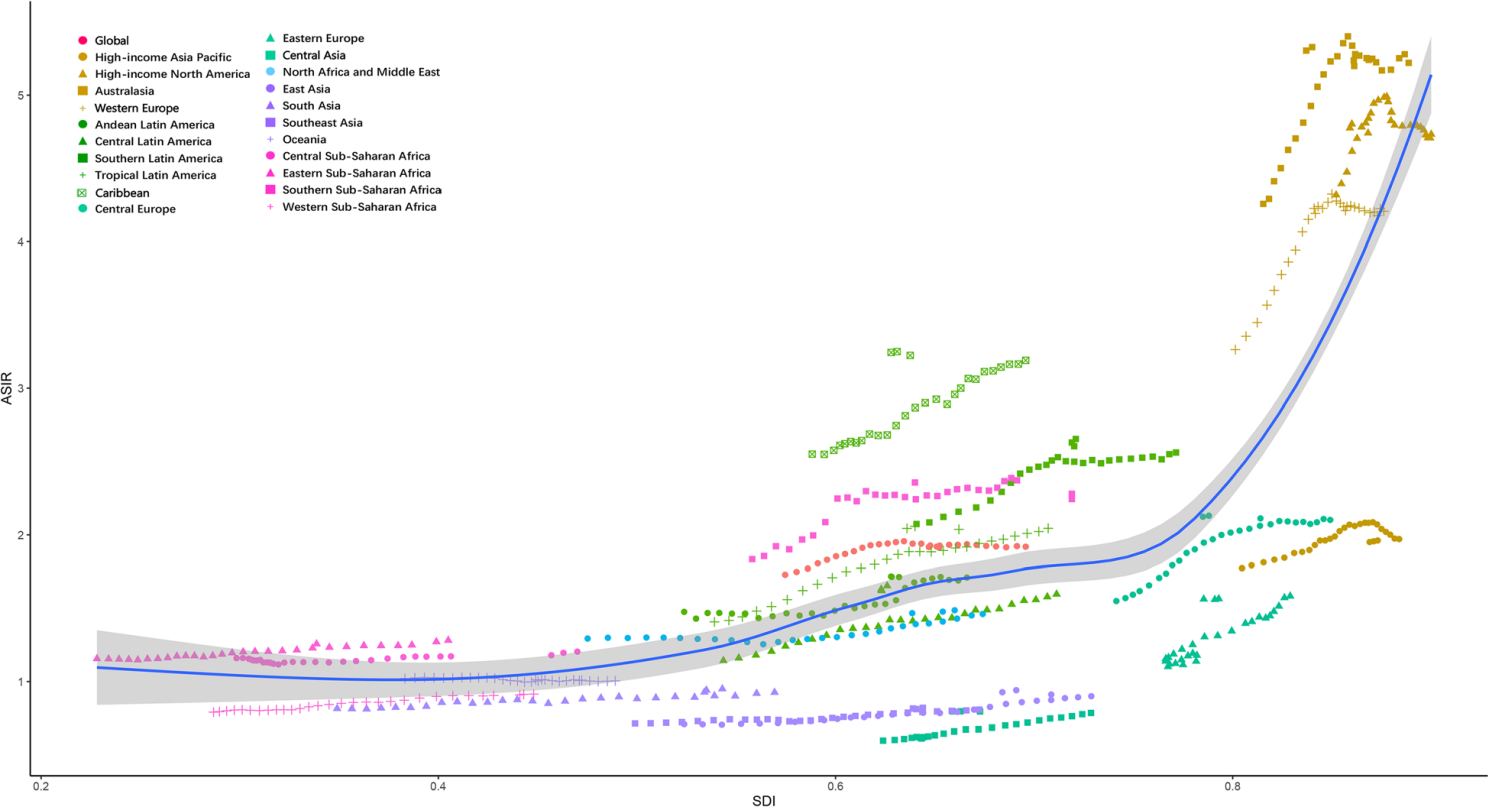
The ASIR of multiple myeloma among regions based on SDI in 2019. ASIR: age standardized incidence rate (per 100,000)

As shown in Table 1, the incident cases of MM increased 1.36 times from 65,940 (95% UI, 155,688–74,058) in 1990 to 155,688 (95% UI, 136,585 – 172,577) in 2019. In 2019, the proportion of cases involving men slightly increased to 54.3% (84,516, 70,924 – 94,910) compared with 50.7% (33,435, 29,581–38,797) in 1990. The ASIR was 1.73/100,000 persons (95% UI, 1.59–1.93) in 1990 and 1.92/100,000 persons (95% UI, 1.68–2.12) in 2019. The increase in the incidence of MM may be attributed to higher diagnosis of MM on population level. The ASIR for men was 1.97/100,000 persons (1.74–2.25) in 1990 and 2.28/100,000 persons (1.91–2.56) in 2019. The ASIR for women was 1.55/100,000 persons (1.40–1.81) in 1990 and 1.62/100,000 persons (1.38–1.83) in 2019. The percentage increase in ASIR from 1990 to 2019 was higher in men (16.03, − 1.45% to 29.18%) than in women (4.65, − 11.58% to 14.6%). Both incident cases (722,250, 62,610 – 82,520) and ASIR (3.77/100,000 persons, 3.29–4.33) of MM were the highest in the high SDI region in 2019, which is higher than that in other SDI regions. Across various countries, ASIR positively correlated with SDI (*ρ* = 0.54, *p* < 0.01). The details are shown in Fig. 1, Supp Fig. 1 and Supp Fig. 2a. SDI also positively (*ρ* = 0.22, *p* = 0.014) correlated with the percentage increase in ASIR from 1990 to 2019 (Supp Fig. 2b).

**Table 1:** Incident cases, deaths and DALYs of multiple myeloma in 2019, and percentage change of age-standardized rates by sex and GBD region

	Incidence			Deaths			DALYs		
	Number of incident cases	Age standardized incidence rate (per 100,000 population)	Percentage change in rates(%), 1990–2019	Number of deaths	Age standardized death rate (per 100,000 population)	Percentage change in rates(%), 1990–2019	Number of DALYs*10^3^	Age standardized DALY rate (per 100,000 population)	Percentage change in rates(%), 1990–2019
Global	155,688 (136585–172,577)	1.92 (1.68–2.12)	10.99(−5.11–21.64)	113,474 (99527–121,735)	1.42 (1.24–1.52)	1.43(− 13.98–7.98)	2497.2 (2190.5–2722.7)	30.26 (26.58–32.9)	−0.85(− 17.48–6.66)
Sex									
Females	71,171 (60343–80,140)	1.62 (1.38–1.83)	4.65(− 11.58–14.6)	53,029 (45149–58,252)	1.21 (1.03–1.33)	−3.82(− 19.80–2.84)	1120.6 (967.7–1243.7)	25.67 (22.15–22.48)	−6.11(− 23.87–1.17)
Males	84,516 (70924–94,910)	2.28 (1.91–2.56)	16.03(− 1.45–29.18)	60,445 (50723–67,056)	1.68 (1.40–1.84)	5.7(− 9.92–14.3)	1376.6 (1150.6–1567.8)	35.51 (29.77–40.03)	3.4(− 14.14–13.72)
Socio-demographic index									
High SDI	72,250 (62610–82,520)	3.77 (3.29–4.33)	16.66 (5.06–32.25)	48,108 (41267–51,245)	2.4 (2.1–2.59)	2.29(− 3.93–11.12)	901.2 (811.9–1007.5)	49.3 (45.25–55.85)	−2.41(− 8.13–13.1)
High-middle SDI	37,329 (30933–41,806)	1.83 (1.52–2.05)	21.54(− 1.95–36.12)	27,224 (22937–29,555)	1.34 (1.13–1.45)	10.66(− 10.62–20.44)	605.9 (506.6–659.7)	29.77 (24.79–32.47)	5.33(− 15.84–15.34)
Middle SDI	25,294 (20919–28,629)	1.01 (0.83–1.14)	19.55(− 14.5–36.97)	21,143 (17470–24,144)	0.86 (0.71–0.99)	11.68(− 21.22–27.39)	134.7 (107.4–158.1)	20.74 (16.96–23.57)	10.48(− 24.71–27.2)
Low-middle SDI	13,654 (11831–15,809)	0.99 (0.86–1.15)	20.83(− 10–38.57)	12,040 (10183–14,016)	0.89 (0.76–1.05)	13.77(− 14.1–29.67)	312.7 (267.2–366.6)	21.75 (18.57–25.5)	14.19(− 14.94–31.05)
Low SDI	5148 (4101–5981)	1 (0.8–1.16)	3.18(− 22.53–22.54)	4970 (3938–5768)	1.01 (0.8–1.17)	5.99(− 21.03–25.9)	540.9 (442.1–614.1)	24.16 (19.2–28.19)	5.2(− 22.32–27.82)
Region									
Andean Latin America	955 (747–1214)	1.71 (1.34–2.18)	16.08(− 25.14–55.8)	788 (622–995)	1.43 (1.13–1.8)	3.46(− 31.28–38.21)	18.9 (14.67–24.07)	33.29 (25.84–42.26)	1.6(− 34.2–38.53)
Australasia	2658 (2101–3388)	5.33 (4.21–6.8)	25.1(− 0.82–60.03)	1541 (1328–1736)	2.97 (2.59–3.39)	6.38(− 4.64–24.37)	29.51 (26.16–34.07)	61.23 (54.51–70.45)	1.41(− 8.47–23.89)
Caribbean	1686 (1401–1993)	3.25 (2.7–3.84)	27.38 (7.33–49.9)	1224 (1022–1442)	2.36 (1.97–2.79)	14.95(− 1.89–34.35)	28.86 (24.02–34.53)	55.51 (46.25–66.5)	16.62(− 0.97–38.02)
Central Asia	608 (533–686)	0.8 (0.7–0.89)	33.1 (12.6–55.55)	502 (441–564)	0.69 (0.61–0.78)	30.58 (11.09–53.2)	14.33 (12.6–16.23)	17.57 (15.43–19.77)	32.24 (12.37–54.72)
Central Europe	4550 (3599–5249)	2.13 (1.69–2.46)	37.47 (6.33–58.98)	3920 (3105–4499)	1.79 (1.42–2.06)	32.93 (3.41–52.72)	83.8 (67.12–96.4)	40.61 (32.49–46.85)	25.67 (2.27–44.11)
Central Latin America	3927 (3269–4645)	1.65 (1.38–1.96)	44.69 (21.08–69.62)	3038 (2529–3589)	1.3 (1.08–1.53)	30.08 (7.57–52.46)	76.73 (62.57–91.25)	31.74 (25.98–37.71)	28.6 (6.83–50.76)
Central Sub-Saharan Africa	630 (390–864)	1.2 (0.74–1.64)	3.84(− 23.17–36.26)	574 (355–783)	1.16 (0.71–1.56)	1.05(− 24.05–29.46)	15.98 (9.98–21.98)	27.62 (17.1–37.65)	0.37(− 26.39–32.62)
East Asia	19,712 (14445–24,287)	0.94 (0.69–1.16)	32.75(− 28.14–71.32)	14,109 (10739–17,150)	0.68 (0.52–0.83)	6.05(− 39.47–35.18)	363.76 (271.11–439.32)	17.25 (12.7–20.81)	6.96(− 42.07–39.48)
Eastern Europe	5358 (4643–6083)	1.57 (1.35–1.78)	42.09 (21.48–60.32)	3986 (3373–4508)	1.15 (0.97–1.31)	31.27 (10.61–49.17)	98.84 (82.51–112.1)	29.54 (24.6–33.43)	27.19 (10.77–44.01)
Eastern Sub-Saharan Africa	2034 (1437–2501)	1.28 (0.91–1.56)	10.84(− 21.33–37.13)	1889 (1348–2308)	1.25 (0.89–1.52)	9.12(− 21.64–35.2)	50.82 (35.96–62.75)	28.94 (20.6–35.64)	6.58(− 25.49–36.05)
High-income Asia Pacific	9191 (7132–10,888)	1.96 (1.58–2.3)	10.62(− 13.64–28.79)	6562 (5165–7282)	1.32 (1.07–1.45)	−3.08(− 21.57–5.56)	113.04 (93.38–123.29)	26.62 (22.07–28.98)	−8.67(− 27.89 - -0.88)
High-income North America	30,394 (26016–36,835)	4.8 (4.12–5.87)	11.22(− 4.48–41.32)	19,942 (18012–22,920)	3.07 (2.8–3.58)	−1.27(− 6.53–21.95)	388.96 (360.5–478.32)	63.18 (58.79–78.56)	−7.85(− 12.97–21.41)
North Africa and Middle East	6373 (4991–7641)	1.49 (1.17–1.77)	14.95(− 23.27–42.18)	5012 (3938–5969)	1.22 (0.96–1.44)	2.91(− 29.84–26.14)	130 (101.74–157.35)	28.4 (22.27–34.07)	0.13(− 33.47–23.52)
Oceania	72 (48–115)	1.01 (0.69–1.55)	− 0.74(− 18.92–23.18)	63 (42–100)	0.94 (0.64–1.43)	−1.9(− 19.53–21.1)	1.83 (1.22–3.02)	22.99 (15.54–36.69)	−2.57(− 21–22.71)
South Asia	13,368 (9787–15,941)	0.95 (0.69–1.13)	16.5(− 18.11–42.11)	11,734 (8615–13,925)	0.86 (0.62–1.02)	8.87(− 24.09–31.24)	305.84 (228.24–362.7)	20.67 (15.34–24.47)	10.05(− 23.54–32.56)
Southeast Asia	5003 (4174–6594)	0.82 (0.68–1.09)	14.96(− 11.79–37.09)	4194 (3495–5587)	0.71 (0.59–0.95)	7.02(− 17.05–25.43)	108.86 (90.98–142.69)	16.9 (14.12–22.27)	4.26(− 21.38–23.88)
Southern Latin America	2212 (1728–2837)	2.66 (2.08–3.41)	28.04(− 2.34–64.15)	1739 (1584–2003)	2.07 (1.88–2.37)	14.63 (1.17–31.88)	38.12 (34.97–44.3)	46.4 (42.61–53.99)	12.67(− 1.76–32.19)
Southern Sub-Saharan Africa	1267 (905–1467)	2.24 (1.6–2.59)	22.37(−3.98–45.5)	1108 (788–1283)	2.04 (1.45–2.35)	20.07(− 4.68–43.35)	29.17 (20.71–33.88)	48.73 (34.63–56.49)	17.56(− 9.33–38.88)
Tropical Latin America	5004 (4350–5394)	2.06 (1.78–2.22)	46.2 (23.92–56.74)	4018 (3400–4313)	1.67 (1.41–1.8)	35.83 (12.05–45.67)	96.79 (85–105.43)	39.11 (34.15–42.37)	28.34 (11.91–36.39)
Western Europe	38,981 (31727–44,710)	4.24 (3.51–4.9)	29.9 (10.88–48.41)	25,996 (21281–27,914)	2.64 (2.22–2.82)	15.62(−2.93–21.83)	461.63 (397.99–495.38)	52.87 (46.52–56.55)	7.97(− 5.58–15.07)
Western Sub-Saharan Africa	1705 (1340–2074)	0.91 (0.72–1.11)	15.57(− 9.48–40.42)	1536 (1189–1882)	0.86 (0.67–1.05)	12.22(− 12.14–35.43)	41.44 (31.75–51.23)	20.47 (15.76–25.1)	11.51(− 13.72–37.56)

**Fig. 2:**
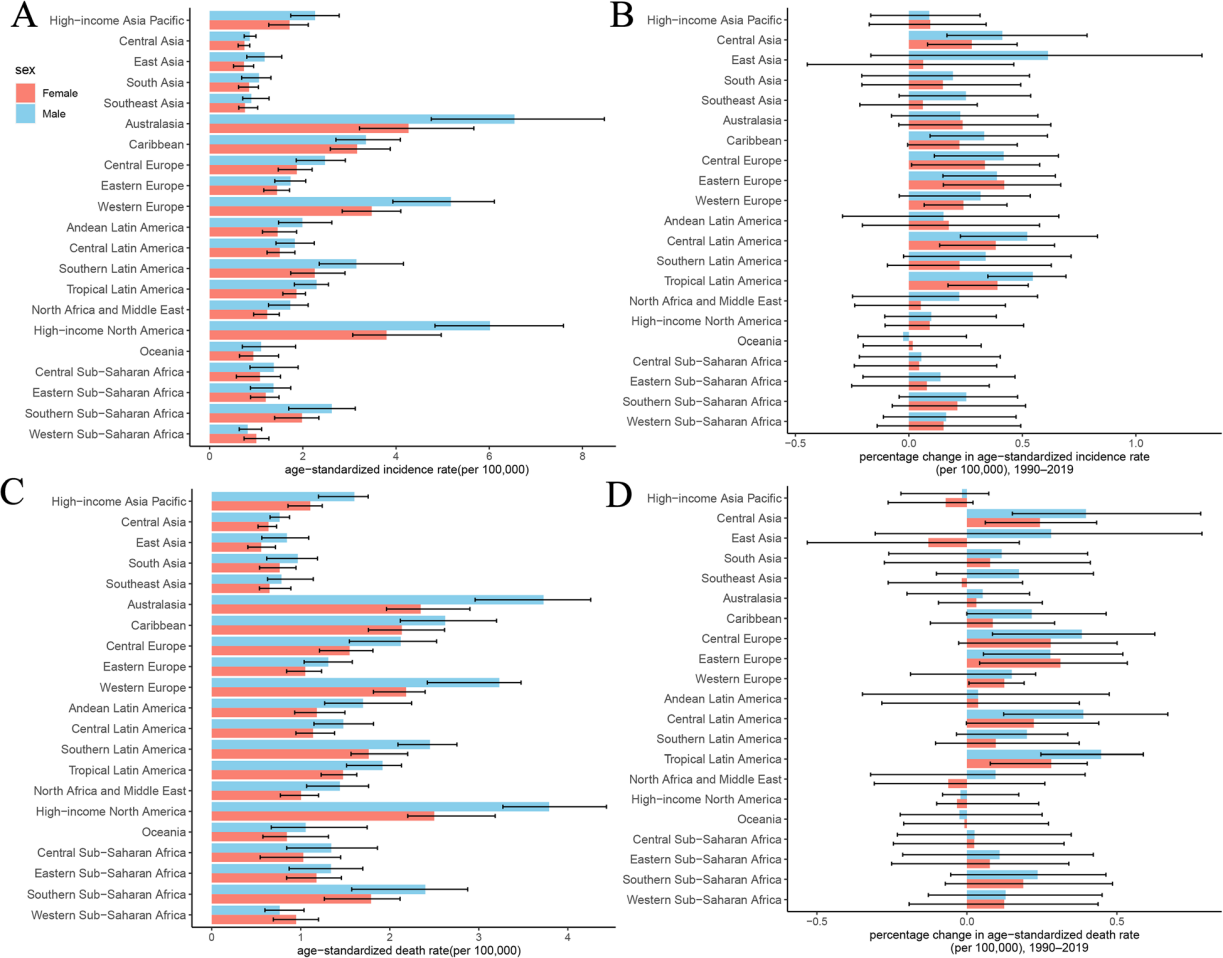
Levels and trends in age-standardized incidence and death rates of multiple myeloma across regions. **a** The age-standardized incidence rates of multiple myeloma in 2019. **b** The percentage change in age-standardized incidence rate of multiple myeloma from 1990 to 2019. **c** The age-standardized death rate of multiple myeloma in 2019. **d** The percentage change in age-standardized death rate of multiple myeloma from 1990 to 2019. GBD = Global Burden of Diseases, Injuries, and Risk Factors Study

**Fig. 3:**
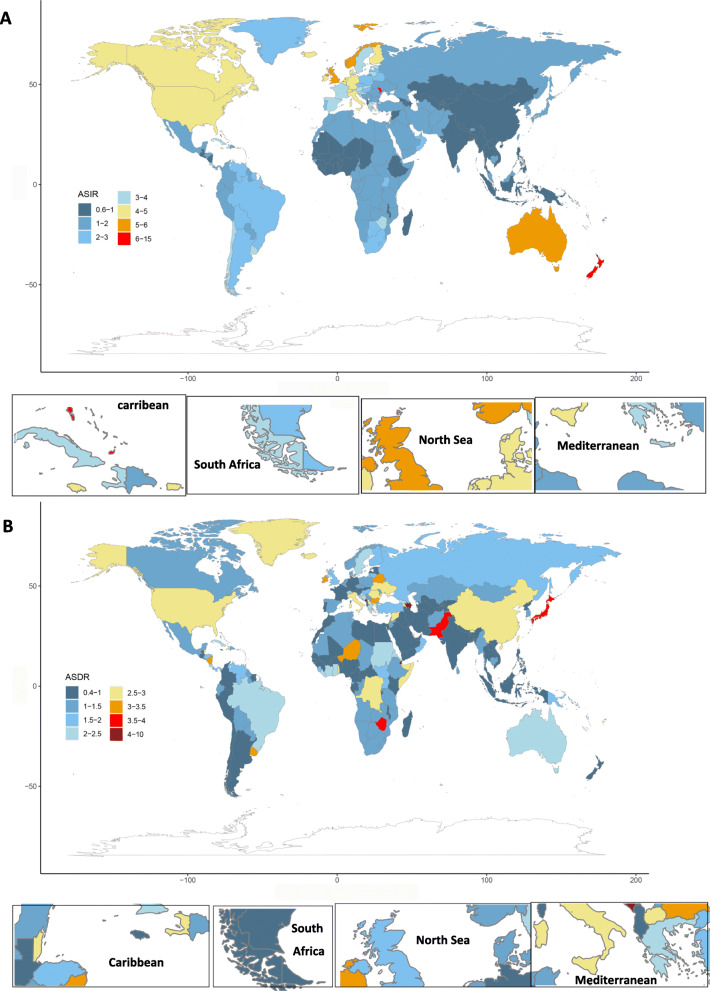
Age-standardized rates of incidence (**a**) and death (**b**) of multiple myeloma worldwide, 2019. ASIR: age standardized incidence rate; ASDR: age standardized death rate

In 2019, the ASIR was the highest in Australasia (5.33/100,000 persons, 4.21–6.8), high-income North America (4.8/100,000 persons, 4.12–5.87), Western Europe (4.24/100,000 persons, 3.51–4.9) and the lowest in Central Asia (0.8/100,000 persons, 0.7–0.89) and Southeast Asia (0.82/100,000 persons, 0.68–1.09) (Fig. 2a). The three regions had the largest increase in ASIR from 1990 to 2019 in Central Latin America, Eastern Europe, and Tropical Latin America. In most areas, the ASIR for men was higher than that for females, except in Western Sub-Saharan Africa. As shown in Fig. 2b, ASIR increased in all GBD (the Global Burden of Disease) regions from 1990 to 2019, except in Oceania. The world map of ASIR and its percentage change in MM are shown in Fig. 3a and Supp Fig. 3a, respectively. As shown in Table 2, the three countries with the highest ASIR were Monaco, Barbados and Dominica in 2019. The number of incident cases of United Arab Emirates (9.85) and Qatar (8.39) had the highest increase times.

**Table 2:** Three countries with the highest and lowest means of incidence, death, or DALYs

Measure	Top three countries			Bottom three countries		
**2019ASR (per 100,000 people)**						
ASIR	Monaco (14.95)	Barbados (8.57)	Dominica (7.25)	Kyrgyzstan (0.62)	Mali (0.66)	Mongolia (0.67)
ASDR	Monaco (9.81)	Barbados (6.17)	Dominica (5.89)	Kyrgyzstan (0.55)	Palau (0.59)	Mongolia (0.60)
Age Standardized DALY Rate	Monaco (199.23)	Barbados (139.63)	Dominica (132.87)	Kyrgyzstan (13.33)	Thailand (14.67)	Palau (14.71)
**Percentage change in rates from 1990 to 2019**						
ASIR	Belarus (1.45)	Jamaica (1.24)	Estonia(1.11)	Bahrain(−0.24)	Northern Mariana Islands(− 0.23)	Guam (− 0.22)
ASDR	Jamaica (1.12)	Belarus (1.07)	Estonia(0.79)	Bahrain(−0.32)	Jordan(−0.28)	Northern Mariana Islands (− 0.52)
Age Standardized DALY Rate	Jamaica (1.18)	Belarus (1.09)	Turkmenistan (0.96)	Bahrain(−0.35)	Jordan(−0.29)	Northern Mariana Islands (− 0.26)
**1990–2019 increase cases times**						
Incidence	United Arab Emirates (9.85)	Qatar (8.39)	Djibouti (4.05)	Tokelau (−0.09)	Niue (−0.01)	Nauru (0.11)
Death	United Arab Emirates (8.63)	Qatar (6.63)	Djibouti(3.33)	Tokelau (−0.15)	Niue (−0.09)	Nauru (0.04)
DALYs	United Arab Emirates (9.35)	Qatar (6.65)	Djibouti (3.75)	Tokelau (−0.12)	Niue (−0.06)	Nauru (0.09)

**Fig. 4:**
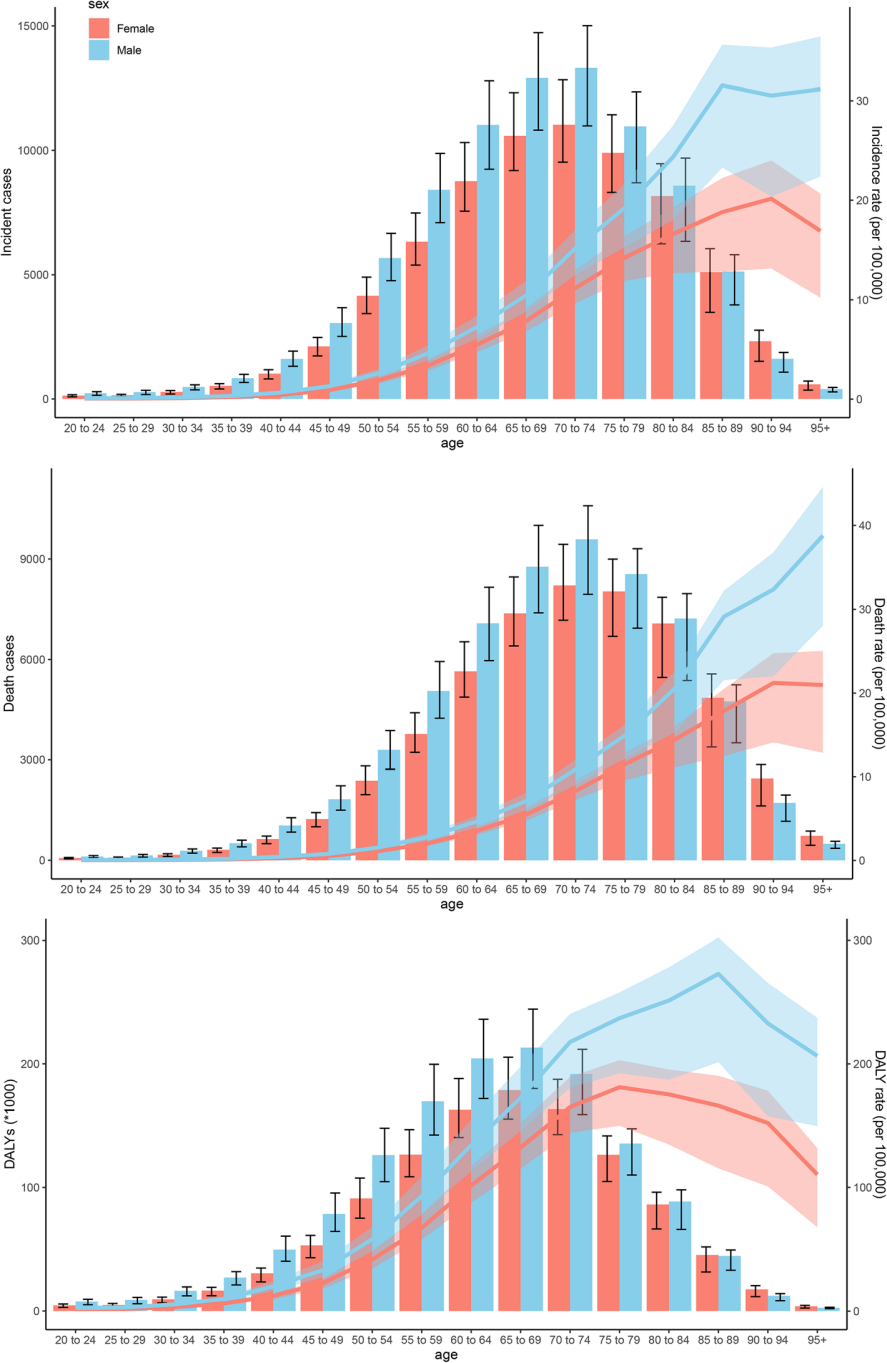
Age-specific counts and rates of multiple myeloma by sex, 2019. **a**: incidence; **b**: death; **c**: DALYs. DALYs: disability-adjusted life-years

**Fig. 5:**
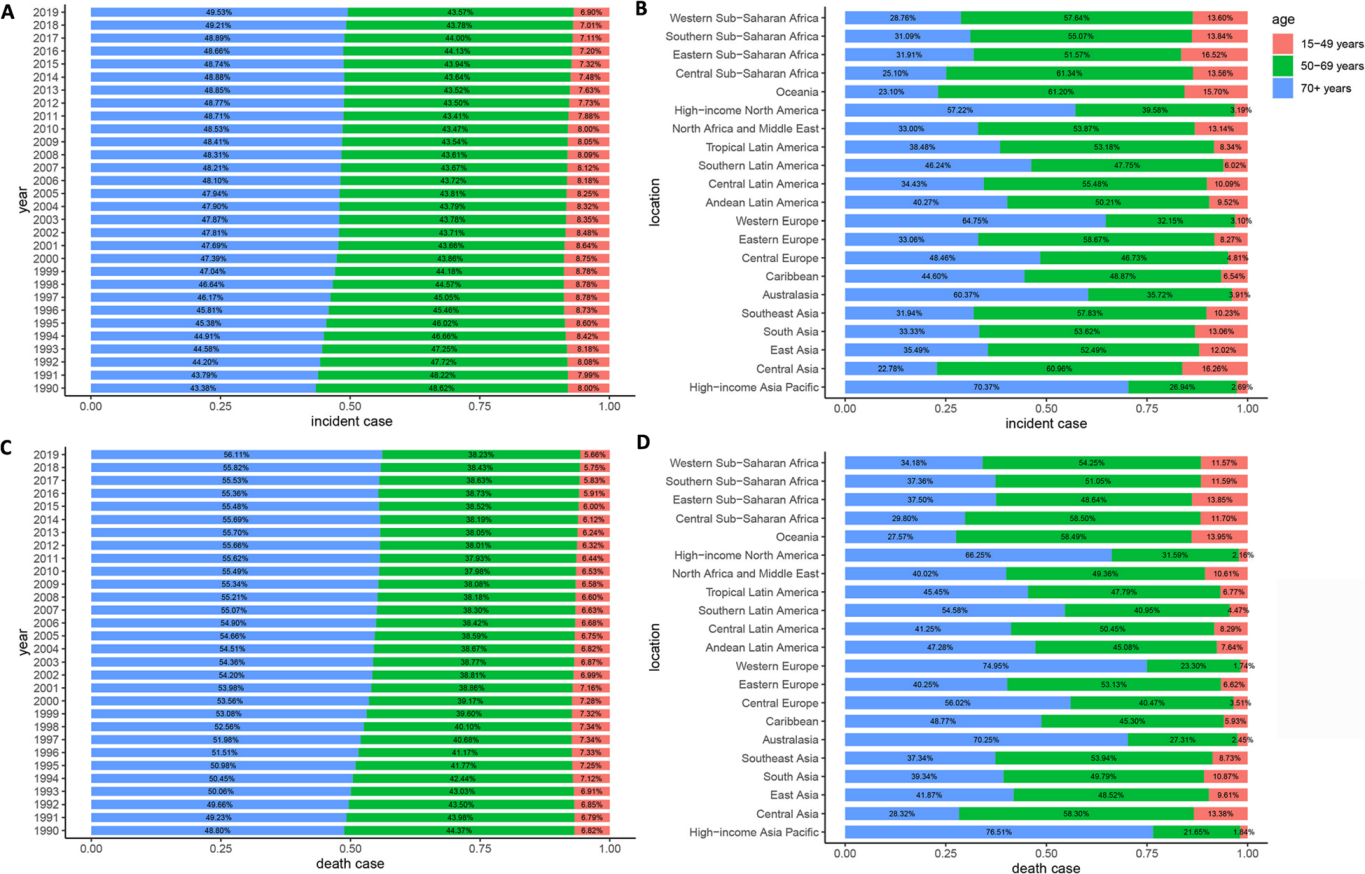
Age distribution of multiple myeloma by years or regions. **a**: by years in incidence cases; **b**: by regions in incidence cases; **c**: by years in death cases; **d**: by regions in death cases

The ASIR for both men and women increased with increasing age, and the ASIR for men was larger than that for women in all age groups (Fig. 4a). The number of incident cases showed a unimodal distribution in men and women, and both peaked at the age of 70–74 years. The median age at diagnosis of MM gradually increased over the past 30 years and was approximately 70 years old (Fig. 5a). In 2019, 6.90, 43.57, and 49.53% of MM patients were < 50, 50–70, and ≥ 70 years, respectively. The proportion of elderly MM patients has gradually increased over the past 30 years. The higher the SDI, the higher the proportion of elderly patients and the lower the proportion of young patients (Fig. 5b). Globally, the ratio of male to female in ASIR peaked at the age of 95+ years with a value of 1.84 (Supp Fig. 4). Generally, the ratio decreased before 75 years and increased after 75 years.

### Mortality burden

**Fig. 6:**
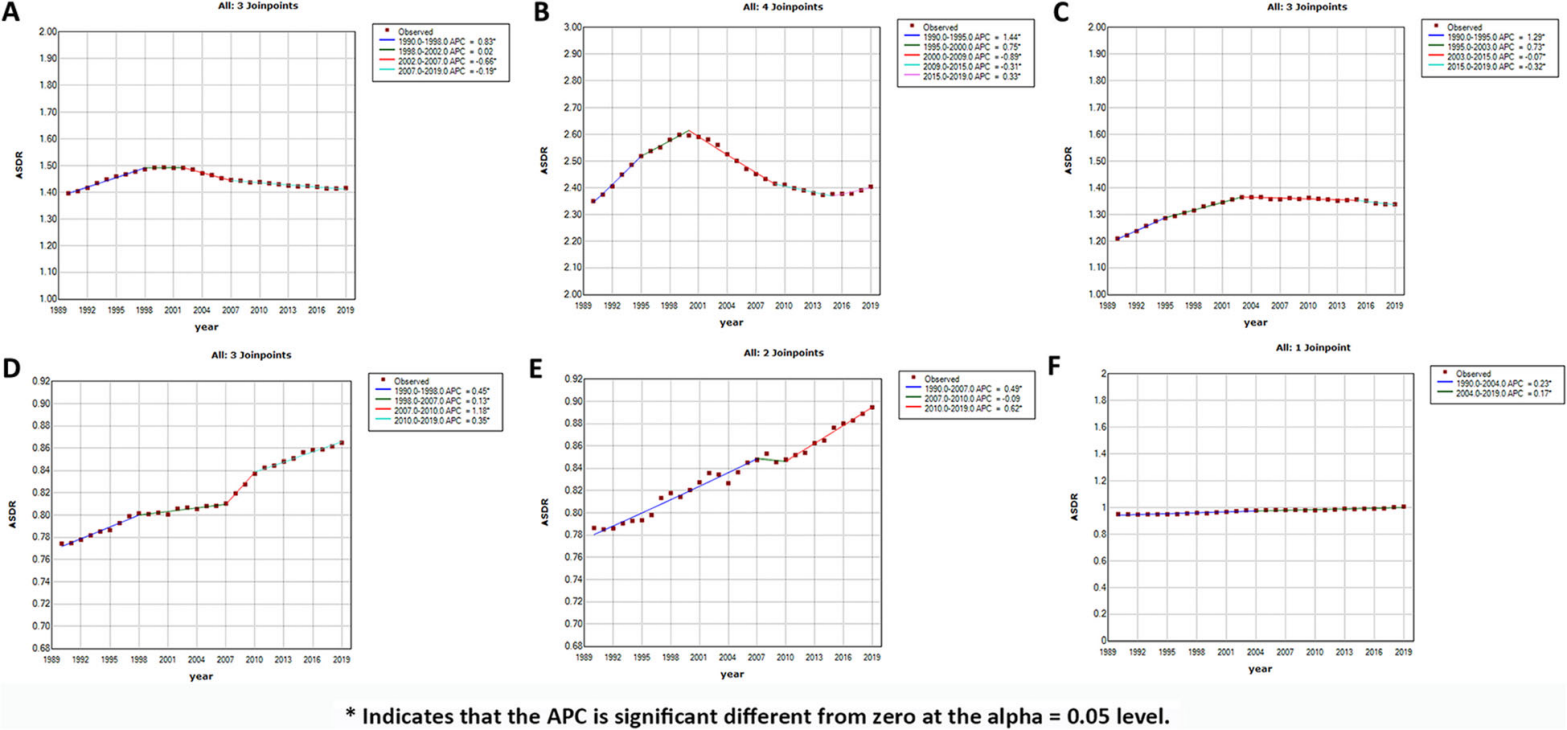
Joinpoint regression analysis of age-standardized death rates (ASDR) among SDI regions from 1990 to 2019. APC: annual percent change. * Indicates that the APC is significant different from zero at the alpha = 0.05 level. **a**: global; **b**: high SDI; **c**: high-middle SDI; **d**: middle SDI; **e**: middle-low SDI; **f**: low SDI. SDI, socio-demographic index. ASDR, age-standardized death rate

As shown in Table 1, the death cases of MM increased from 51,862 (95% UI, 47,710–58,980) in 1990 to 113,474 (95% UI, 99,527 – 121,735) in 2019. The ASDR was 1.39/100,000 persons (95% UI, 1.28–1.58) in 1990 to 1.42/100,000 persons (95% UI, 1.24–1.52) in 2019. In 2019, the number of male deaths and ASDR for men were 60,445 (50,723 – 67,056) and 1.68/100,000 persons (1.40–1.84), respectively. The number of female deaths and ASDR for women were 53,029 (45,149 – 58,252) and 1.21/100,000 persons (1.03–1.33), respectively. The percentage change in the ASDR from 1990 to 2019 was 5.7% (− 9.92–14.3%) for men and − 3.82% (− 19.80–2.84%) for women. To further clarify the trend of ASDR in men and women, joinpoint regression model was conducted (Supp Fig. 5a and Supp Fig. 5b). In recent 15 years, ASDR showed a steady trend among men, and a downward trend among women. Both deaths (48,108, 41,267 – 51,245) and the ASDR (2.4/100,000 persons, 2.1–2.59) of MM were the highest in the high SDI region in 2019. In addition, the ASDR positively correlated with SDI (*ρ* = 0.46, *p* < 0.01); the details are shown in Supp Fig. 6 and Supp Fig. 2c. Globally, the ASDR showed a slow upward trend before 1998, a downward trend from 2002 to 2007, and a slow downward over the past 12 years. This trend was more obvious in the high SDI region (Fig. 6). In middle and middle-low SDI regions, the mortality showed an obvious upward trend. In 2019, the three countries with the highest ASDR were Monaco, Barbados and Dominica and the number of death cases of United Arab Emirates (8.63) and Qatar (6.63) had the highest increase times. In 2019, the three GBD regions with the highest ASDR were Australasia, high-income North America, and Western Europe **(**Fig. 2c). The world map of the ASDR in 2019 and the percentage change in MM from 1990 to 2019 are shown in Fig. 2d, Fig. 3b and Supp Fig. 3b, respectively.

The ASDR for both men and women increased with increasing age and was larger for men than women in all age groups (Fig. 4b). The number of death cases showed a unimodal distribution in men and women and that peaked at 70–74 years. The median age at death attributable to MM was approximately 75 years (Fig. 5c). The proportion of age group of death cases was similar to that of incident cases (Fig. 5d). Globally, the ratio of male to female in the ASDR was also similar to that of ASIR (Supp Fig. 7).

### DALYs burden

As shown in Table 1, the DALYs of MM increased from 1,223,362 (95% UI, 1,122,712–1,412,932) in 1990 to 2,497,205 (95% UI, 2,190,467–2,722,668) in 2019. The age-standardized DALY rate was 30.52/100,000 persons (95% UI, 27.98–35.00) in 1990 and 30.26/100,000 persons (95% UI, 26.58–32.9) in 2019. In 2019, the number of DALYs and age-standardized DALY rate for women were 1,120,581 (967,699–1,243,740) and 25.67/100,000 persons (22.15–22.48), respectively. The number of DALYs and age-standardized DALY rate for men were 1,376,624 (1,150,624–1,567,825) and 35.51/100,000 persons (29.77–40.03), respectively. In addition, age-standardized DALY rates positively correlated with SDI (Supp Fig. 8). The age-standardized DALY rate showed a unimodal distribution in men and women and that for men peaked at 85–89 years and for women at 75–79 years (Fig. 4c).

### Risk factors attributable to MM burden

**Fig. 7:**
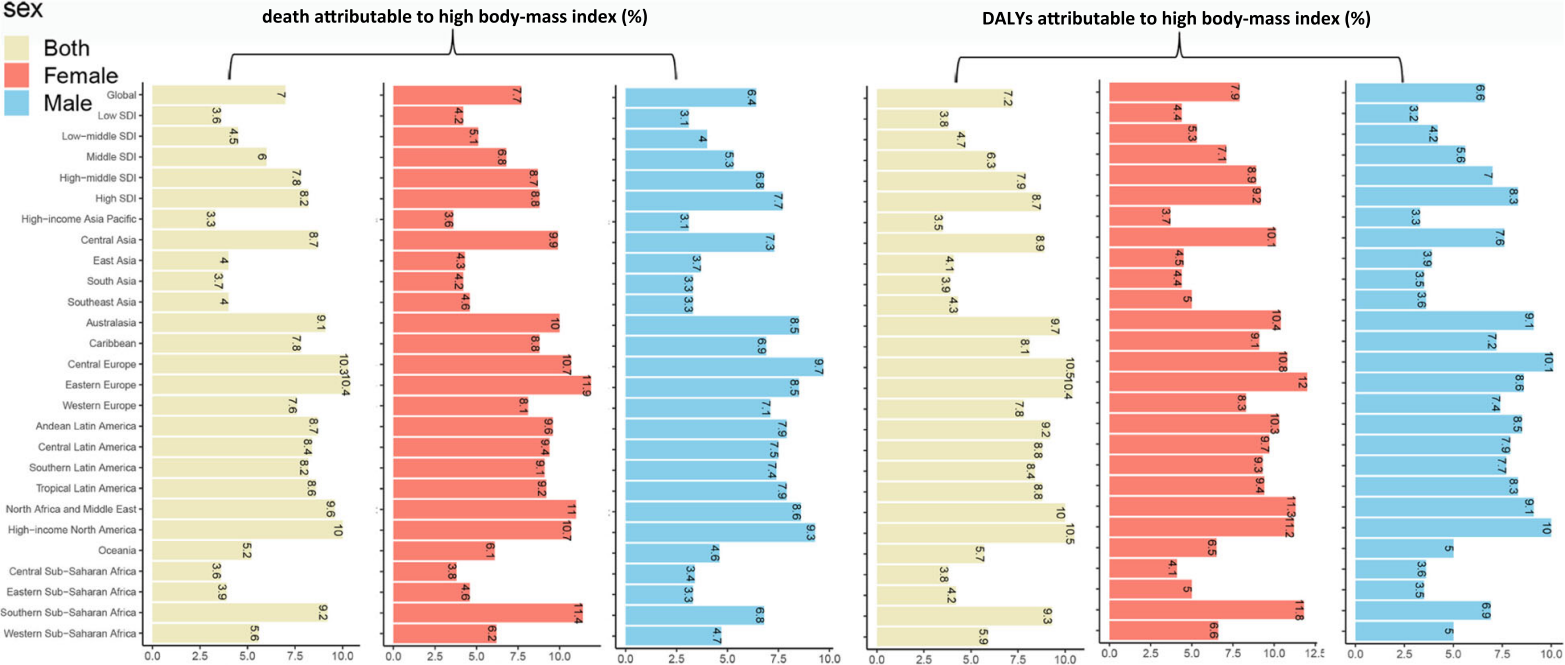
The percentage of age-standardized deaths and DALYs attributable to high body-mass index by regions, 2019

The percentage of age-standardized deaths attributable to a high body mass index in each region is shown in Fig. 7. Globally, the percentage of age-standardized death and DALYs attributable to a high body mass index has increased over the past 30 years (Supp Fig. 9). In 2019, the percentage was 7.0% (95% UI, 3.1–12.5%), which was somewhat higher for women (7.7%) than for men (6.4%). In southern Sub-Saharan Africa, the percentage for women was 11.4%, nearly double compared with that for men (6.8%). Similar patterns of the DALYs attributable to a high body mass index were observed in GBD regions. The percentage of age-standardized DALYs attributable to a high body mass index was 7.17% (95% UI, 3.18–12.47%), which was slightly higher for women (7.86%) than for men (6.59%).

## Discussion

To our knowledge, this study was the latest assessment of the global burden of MM based on Global Burden of Disease Study 2019. In recent decades, epidemiological studies of MM have mainly focused on individuals and several countries [12–16]. The latest study was conducted based on Global Burden of Disease Study 2016 [17]. The study was highly significant and evaluated the association between stem-cell transplant, lenalidomide and bortezomib approval and the decreasing trend in high SDI regions. We updated the data of global burden of MM based on Global Burden of Disease Study 2019 and added the more detailed information of age, sex, SDI and risk factor. Our results revealed that the increasing global burden may continue with population aging, whereas mortality may continue to decrease with the progression of medical technology. MM had the highest ASIR and ASDR in high SDI region in 2019, especially in Australasia, North America, and Western Europe. These findings were consistent with that in 2016. In addition, Monaco had the highest ASIR and ASDR in 2019, which was almost half as high as the second highest country Barbados. Understanding why Monaco had so high incidence and mortality may be significant to explore the risk factors for MM. Over the past 30 years, the number of incident and death cases of United Arab Emirates and Qatar had the largest growth multiple, which was more than twice that of all other countries. The reason for this huge growth multiple was also worth exploring. In the Global Cancer Incidence, Mortality and Prevalence project, the reported new cases were 159,985 and death cases were 106,105 [1]. Although our estimates differ slightly from their estimates, this may be owing to differences in data sources and estimation methods [18].

The burden pattern of MM was diverse in different age groups and sexes. The SEER database has reported that approximately 99% of patients were diagnosed with MM at ≥40 years, and the incidence increased with age. Other studies also showed a higher incidence in men; the total proportion of men was reportedly 51% [19–21]. Our study also suggested that the incidence and mortality of men were slightly higher than those of women and increased with increasing age. Furthermore, we found that mortality of men was stable, while that of women was declining over the past 15 years. These findings indicated that the difference in mortality between men and women would further increase. The men to female ratio of incidence and mortality peaked at 25–29 years and 95+ years. This phenomenon has not been completely clarified. In addition, the incidence in men and women further increased and mortality decreased in women and increased in men over the past 30 years. The median age at diagnosis and death of MM was approximately 70 years and 75 years, respectively. In 2019, the proportion of incident cases and death cases in those aged ≥70 years increased to 49.53 and 56.11%, respectively, which may be due to the aging population.

ASIR and ASDR were 2–3 times higher in high SDI than in other SDI regions. Several previous studies showed no significant association between SDI and the rate of MM [22, 23]. Another study indicated that the rate of MM had a possible positive link with SDI [24]. Compared with the small sample-sized studies conducted decades earlier (less than 200 patients), another study, published in 2015, assessed 562 patients diagnosed with MM at the authors’ institution and 45,505 patients with MM from the SEER database; they found that the rate of MM and SDI value positively correlated [25]. This provides strong support for our conclusions. Moreover, the higher the SDI value, the more the MM age distribution tended to be aging. It may be associated with the aging population worldwide and the increased exposure to risk factors, such as a high body mass index.

Our study suggests that the percentage of a high body mass index-related MM increased annually. The percentage of women was higher than that of men and had a positive association with SDI. With societal development, this percentage will further increase. Excess body weight has gradually become a serious threat worldwide, and it is a risk factor for several cancer types [26]. A meta-analysis indicated that overweight and obese people had a 12 and 27% increased risk of MM, respectively [27]. Another study also suggested that being overweight was a risk factor for MM [28]. The mechanism of the link between being overweight and MM remains unclear and may be explained by the following hypothesis. The occurrence of MM is closely related to the bone marrow microenvironment, and MM cells depend on the regulation of other surrounding cells, such as adipocytes [29, 30]. Reportedly, interleukin-6 (IL-6) is an effective growth factor for MM, and its level can reflect the patient’s prognosis [31]. In overweight people, the level of IL-6 increases, some of which can be produced by adipocytes [32]. Moreover, adiponectin, another inflammatory mediator secreted by fat cells, is negatively correlated with body weight, which can reduce the risk of MM [32, 33]. High insulin-like growth factor-1 (IGF-1) in obese people has also been shown to inhibit MM cell apoptosis and induce MM cell proliferation [34].

Globally, Australasia, high-income North America, and Western Europe had the highest ASIR and ASDR. Interestingly, the ASDR increased during 1990–1998, decreased obviously during 2002–2007, and decreased slowly after 2007 worldwide. This downward trend was more obvious in high SDI region. With progress in auto-HSCT and launch of new agents since the early twenty-first century, the survival of MM patients has been significantly prolonged, which may contribute to the decline in mortality observed during 2001–2007 [35]. In recent years, the development of chimeric antigen receptor T cell technology provided strong support for further reducing the mortality of MM [36]. This encouraging phenomenon indicated that with the progression of medical technology, it was possible to continuously reduce the mortality and even completely conquer MM.

There were some unavoidable limitations in this study. At first, there was only one risk factor (high body mass index) for MM in the database; we lacked information on family genetic history, gene mutation factors, chronic pancreatitis factors, diet, endocrine risk factors and other factors. We cannot evaluate the influence of these risk factors on MM. Second, this study did not examine the modern therapies for 21 world regions and 204 countries and territories from 1990 to 2019 and explored the association between modern therapies and the trend of MM burden. Third, comparisons require summary metrics in the form of standardized estimates that allow significant juxtaposition of non-fatal health outcomes (for example, years lived with disability) and deaths. As our epidemiological knowledge is limited due to various reasons, the width of 95% UI provides a mechanism of communicating to users the limitations of estimates for different diseases, injuries, and risk factors [37]. Without these estimates, there would be no GBD studies.

## Conclusions

The number of incident cases and death cases of MM in 2019 was more than double than those in 1990. The increasing global burden may continue with population aging, whereas decreasing mortality may continue with the progression of medical technology. In recent 15 years, ASDR showed a steady tendency for men, and a downward tendency for women. Incidence and mortality were higher in countries with high SDI, especially in Australasia, North America, and Western Europe. The percentage of age-standardized death and DALYs attributable to a high body mass index increased over the past 30 years. These findings should be considered for formulating specific local strategies to reduce the burden of MM.

## Supplementary Information

**Additional file 1: Supp Fig. 1.** The change trends of age-standardized incidence, death and DALYs rate among different SDI quintiles. **Supp Fig. 2**. The correlation between SDI and age-standardized rates and percentage change. **a**: ASIR; **b**: percentage change in incidence; **c**: ASDR; **d**: percentage change in death rate. The circles represent countries that were available on SDI data. The size of circle represents the number of multiple myeloma patients. The ρ indices Person correlation coefficient and *p* values were derived from Pearson correlation analysis. ASIR: age-standardized incidence rate; ASDR: age-standardized death; SDI, socio-demographic index. **Supp Fig. 3**. The percentage change of multiple myeloma in age-standardized rates worldwide in 2019. **a**: incidence; **b**: death rate. **Supp Fig. 4**. The ratio of male to female ASIR among different age groups in 1990 and 2019. **a**: global; **b**: high SDI; **c**: high-middle SDI; **d**: middle SDI; **e**: middle-low SDI; **f**: low SDI. SDI, socio-demographic index. ASIR, age-standardized incidence rate. **Supp Fig. 5**. Joinpoint regression analysis of age-standardized death rates (ASDR) among males and females from 1990 to 2019. APC: annual percent change. **a**: male; **b**: female. * Indicates that the APC is significant different from zero at the alpha = 0.05 level. ASDR, age-standardized death rate. **Supp Fig. 6**. The ASDR of multiple myeloma among regions based on SDI in 2019. ASDR: age standardized death rate (per 100,000 population). **Supp Fig. 7**. T The ratio of male to female ASDR among different age groups in 1990 and 2019. **a**: global; **b**: high SDI; **c**: high-middle SDI; **d**: middle SDI; **e**: middle-low SDI; **f**: low SDI. SDI, socio-demographic index. ASDR, age-standardized death rate. **Supp Fig. 8**. The age-standardized DALYs rate of multiple myeloma among regions based on SDI in 2019. **Supp Fig. 9**. The percentage of age-standardized deaths and DALYs attributable to high body-mass index from 1990 to 2019.

## Data Availability

The datasets generated and/or analysed during the current study are available in the Global Health Data Exchange query tool (http://ghdx.healthdata.org/gbd-results-tool). Data can be used, shared, modified, or built upon by non-commercial users.

## Abbreviations

SDI: social-demographic index
DALYs: disability adjusted life-years
ASIR: age-standardized incidence rate
ASDR: age-standardized death rate
ASR: age-standardized incidence/death/DALY rate
GBD: The Global Burden of Disease
MM: multiple myeloma
auto-HSCT: autologous hematopoietic stem cell transplantation
