# Supplementary material for: Measuring the global, regional, and national burden of multiple myeloma from 1990 to 2019
Source: BMC Cancer. 2021 May 25;21:606. doi: 10.1186/s12885-021-08280-y (PMC8152089; doi:10.1186/s12885-021-08280-y)
Supplement: Supplementary file 1 — Additional file 1: Supp Fig. 1. The change trends of age-standardized incidence, death and DALYs rate among different SDI quintiles. Supp Fig. 2. The correlation between SDI and age-standardized rates and percentage change. a: ASIR; b: percentage change in incidence; c: ASDR; d: percentage change in death rate. The circles represent countries that were available on SDI data. The size of circle represents the number of multiple myeloma patients. The ρ indices Person correlation coefficient and p values were derived from Pearson correlation analysis. ASIR: age-standardized incidence rate; ASDR: age-standardized death; SDI, socio-demographic index. Supp Fig. 3. The percentage change of multiple myeloma in age-standardized rates worldwide in 2019. a: incidence; b: death rate. Supp Fig. 4. The ratio of male to female ASIR among different age groups in 1990 and 2019. a: global; b: high SDI; c: high-middle SDI; d: middle SDI; e: middle-low SDI; f: low SDI. SDI, socio-demographic index. ASIR, age-standardized incidence rate. Supp Fig. 5. Joinpoint regression analysis of age-standardized death rates (ASDR) among males and females from 1990 to 2019. APC: annual percent change. a: male; b: female. * Indicates that the APC is significant different from zero at the alpha = 0.05 level. ASDR, age-standardized death rate. Supp Fig. 6. The ASDR of multiple myeloma among regions based on SDI in 2019. ASDR: age standardized death rate (per 100,000 population). Supp Fig. 7. T The ratio of male to female ASDR among different age groups in 1990 and 2019. a: global; b: high SDI; c: high-middle SDI; d: middle SDI; e: middle-low SDI; f: low SDI. SDI, socio-demographic index. ASDR, age-standardized death rate. Supp Fig. 8. The age-standardized DALYs rate of multiple myeloma among regions based on SDI in 2019. Supp Fig. 9. The percentage of age-standardized deaths and DALYs attributable to high body-mass index from 1990 to 2019. [file 12885_2021_8280_MOESM1_ESM.pdf]

Measuring the global, regional, and national burden of multiple myeloma from 1990 to 2019

Linghui Zhou[#] [1,2,3,4], Qin Yu[#] [5], Guoqing Wei[1,2,3,4], Linqin Wang[1,2,3,4], Yue Huang[1,2,3,4], Kejia Hu[1,2,3,4], Yongxian Hu[1,2,3,4], He Huang[1,2,3,4]

1. Bone Marrow Transplantation Center, the First Affiliated Hospital, Zhejiang University School of Medicine;
2. Institute of Hematology, Zhejiang University;
3. Zhejiang Province Engineering Laboratory for Stem Cell and Immunity Therapy;
4. Zhejiang Laboratory for Systems & Precision Medicine, Zhejiang University Medical Center;
5. College of Life Science, Zhejiang Chinese Medical University, Hangzhou, China;

# Contributed equally.

Corresponding author:

Yongxian Hu, MD/PhD

Bone Marrow Transplantation Center, The First Affiliated Hospital, School of Medicine, Zhejiang University. No.79 Qingchun Road, Hangzhou, China.

Telephone and Fax: 86-0571-87236706 E-mail: 1313016@zju.edu.cn

He Huang, Professor/MD/PhD

Bone Marrow Transplantation Center, The First Affiliated Hospital, School of Medicine, Zhejiang University. No.79 Qingchun Road, Hangzhou, China.

Telephone and Fax: 86-0571-87236706 E-mail: huanghe@zju.edu.cn

Supp Figures

Supp Figure 1 The change trends of age-standardized incidence, death and DALYs rate among different SDI quintiles.

Supp Figure 2 The correlation between SDI and age-standardized rates and percentage change. A: ASIR; B: percentage change in incidence; C: ASDR; D: percentage change in death rate. The circles represent countries that were available on SDI data. The size of circle represents the number of multiple myeloma patients. The ρ indices Person correlation coefficient and p values were derived from Pearson correlation analysis. ASIR: age-standardized incidence rate; ASDR: age-standardized death; SDI, socio-demographic index.

Supp Figure 3 The percentage change of multiple myeloma in age-standardized rates worldwide in 2019. A: incidence; B: death rate

Supp Figure 4 The ratio of male to female ASIR among different age groups in 1990 and 2019. A: global; B: high SDI; C: high-middle SDI; D: middle SDI; E: middle-low SDI; F: low SDI. SDI, socio-demographic index. ASIR, age-standardized incidence rate

Supp Figure 5 Joinpoint regression analysis of age-standardized death rates (ASDR) among males and females from 1990 to 2019. APC: annual percent change. A: male; B: female. * Indicates that the APC is significant different from zero at the alpha = 0.05 level. ASDR, age-standardized death rate.

Supp Figure 6 The ASDR of multiple myeloma among regions based on SDI in 2019. ASDR: age standardized death rate (per 100,000 population).

Supp Figure 7 T The ratio of male to female ASDR among different age groups in 1990 and 2019. A: global; B: high SDI; C: high-middle SDI; D: middle SDI; E: middle-low SDI; F: low SDI. SDI, socio-demographic index. ASDR, age-standardized death rate

Supp Figure 8 The age-standardized DALYs rate of multiple myeloma among regions based on SDI in 2019.

Supp Figure 9 The percentage of age-standardized deaths and DALYs attributable to high body-mass index from 1990 to 2019.

Correspondence with GBD Management Team (gbdsec@uw.edu)

Supp Figure 1 The change trends of age-standardized incidence, death and DALYs rate among different SDI quintiles.

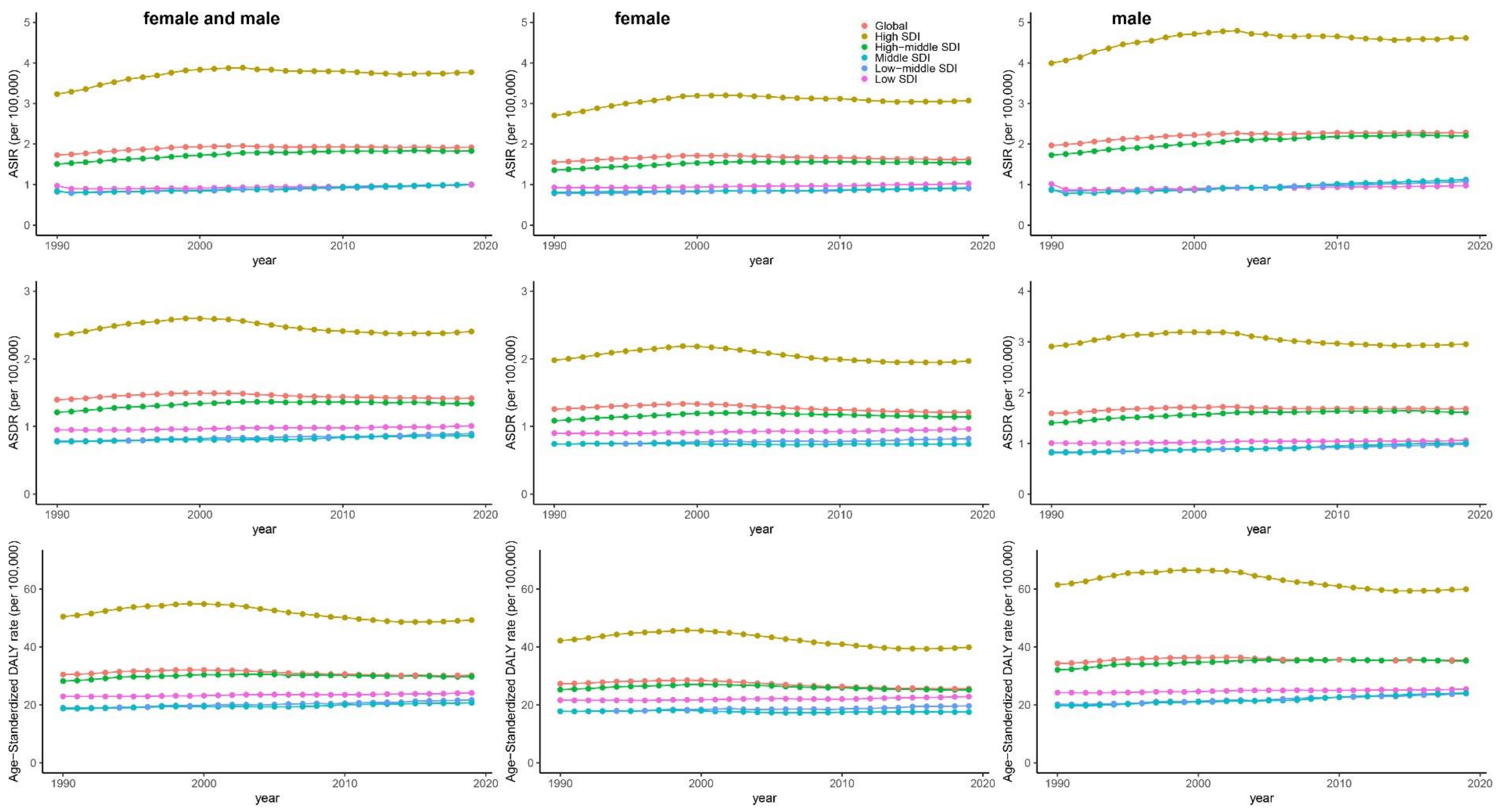

Supp Figure 2 The correlation between SDI and age-standardized rates and percentage change. A: ASIR; B: percentage change in incidence; C: ASDR; D: percentage change in death rate. The circles represent countries that were available on SDI data. The size of circle represents the number of multiple myeloma patients. The ρ indices Person correlation coefficient and p values were derived from Pearson correlation analysis. ASIR: age-standardized incidence rate; ASDR: age-standardized death; SDI, socio-demographic index.

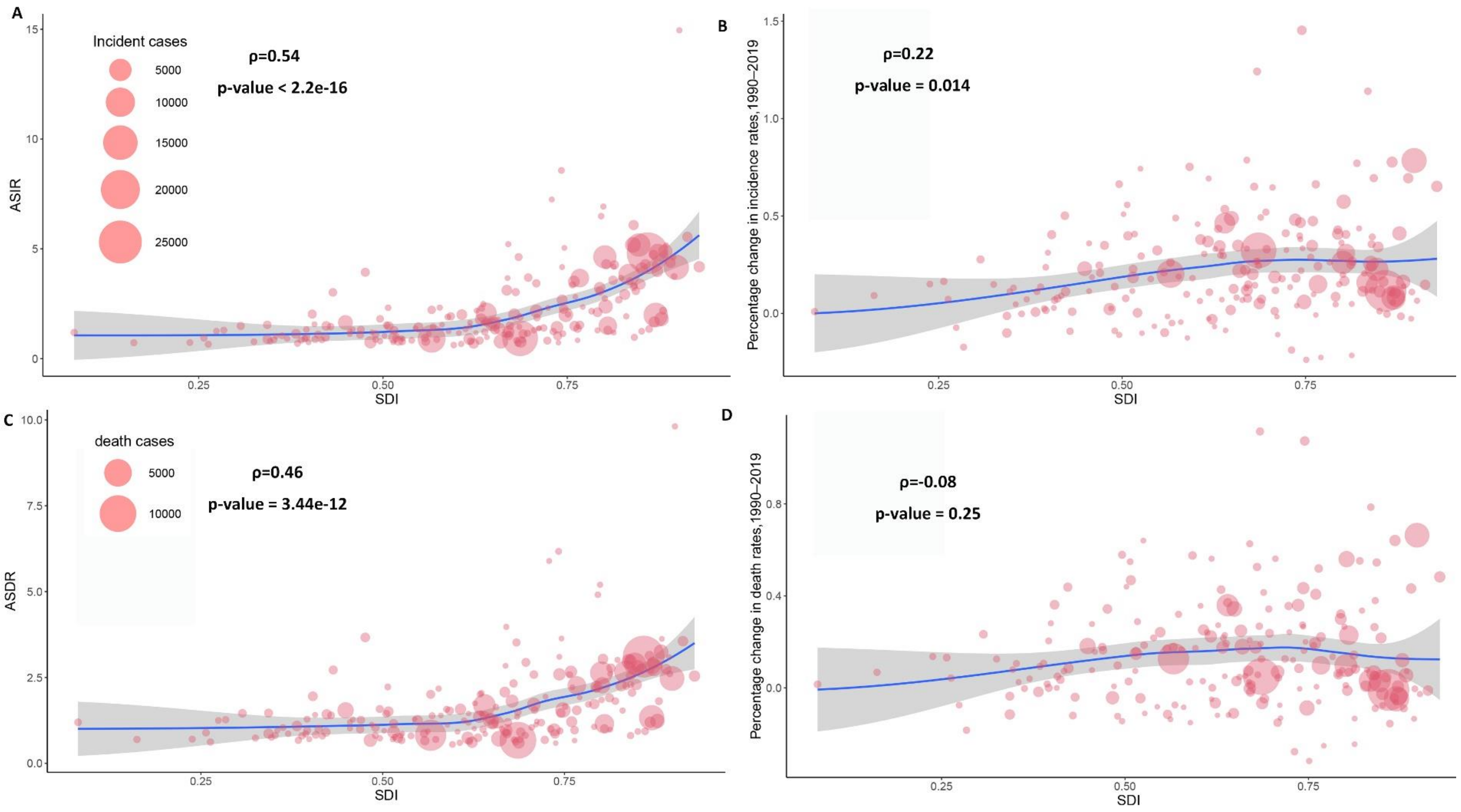

Supp Figure 3 The percentage change of multiple myeloma in age-standardized rates worldwide in 2019. A: incidence; B: death rate

A

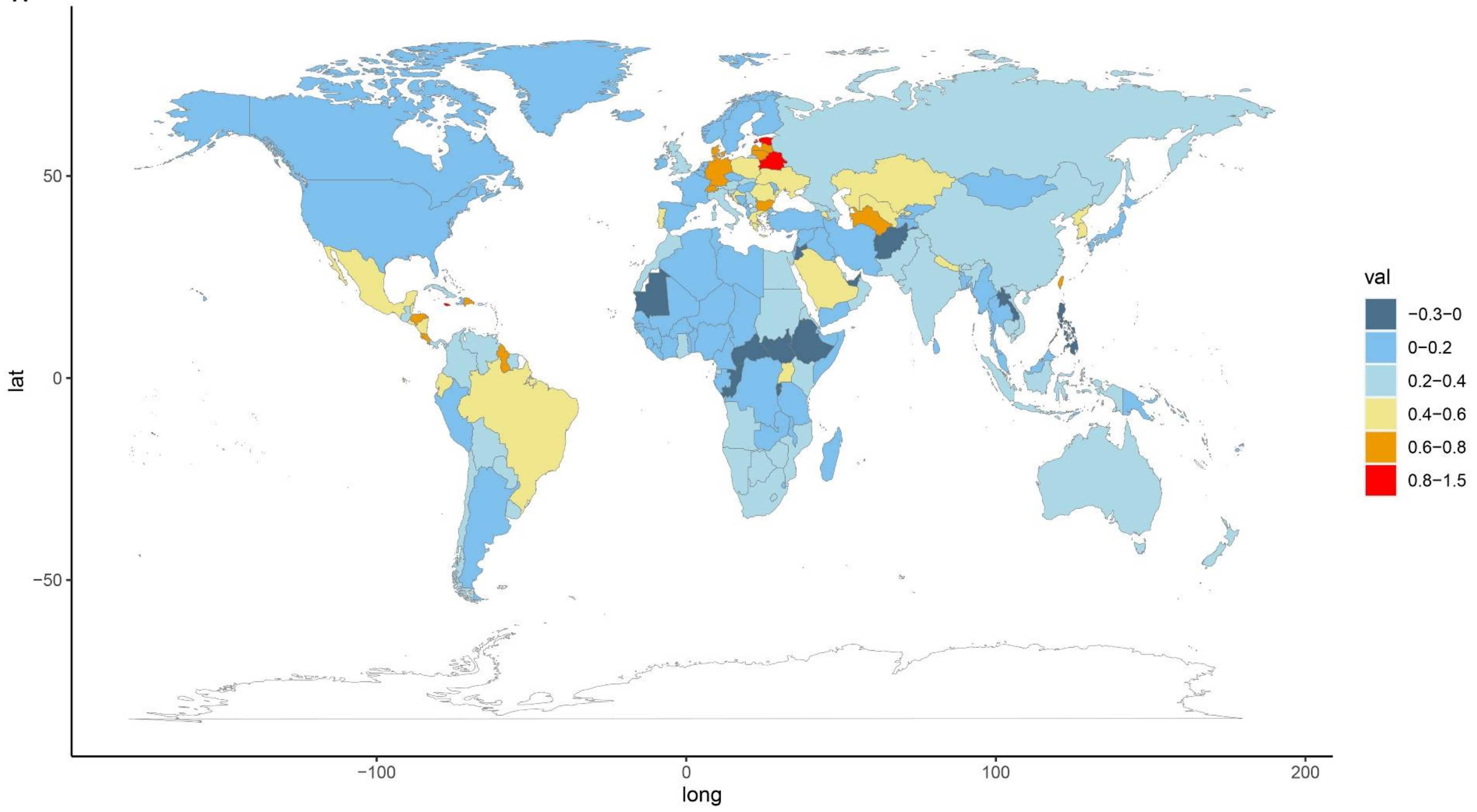

B

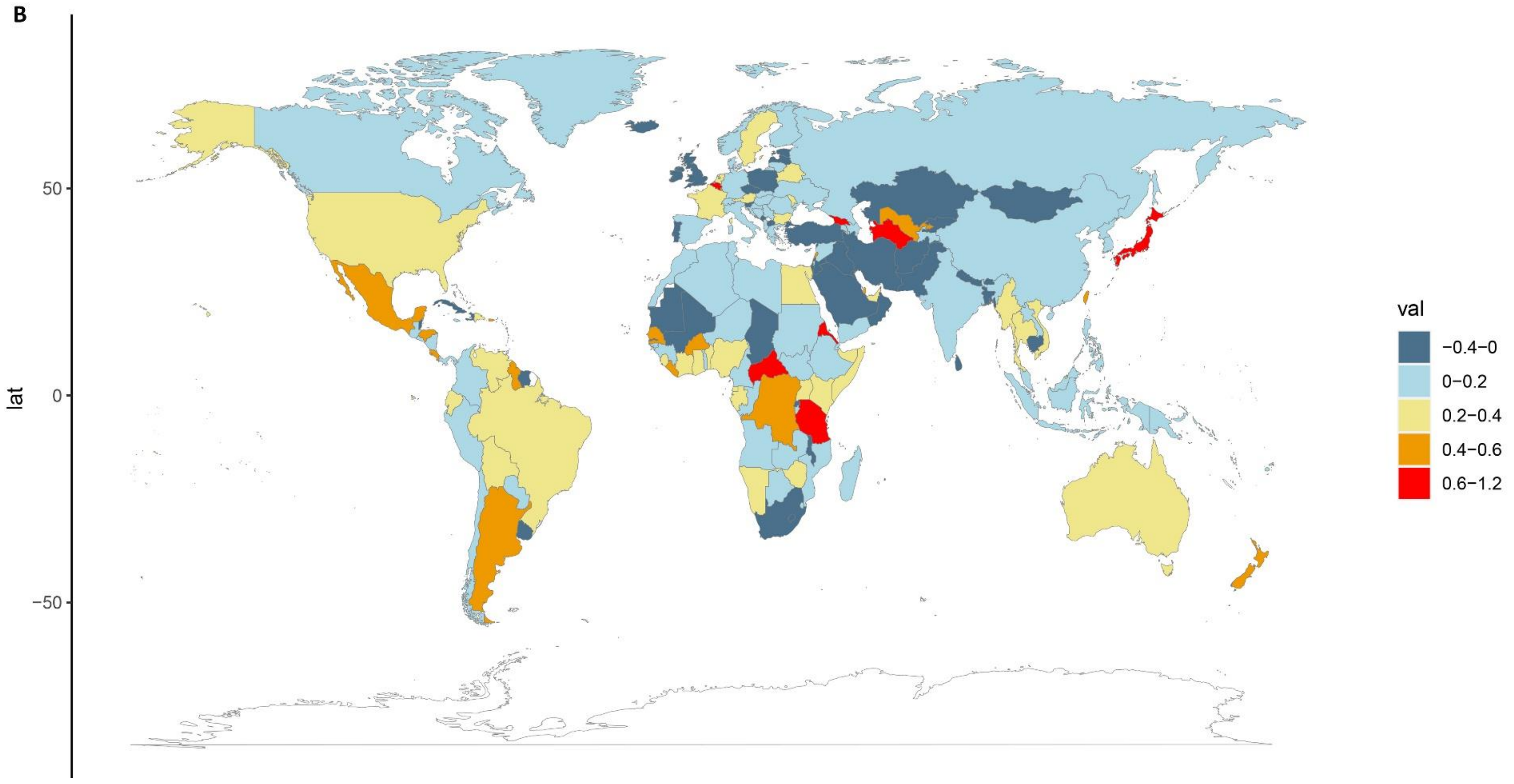

Supp Figure 4 The ratio of male to female ASIR among different age groups in 1990 and 2019. A: global; B: high SDI; C: high-middle SDI; D: middle SDI; E: middle-low SDI; F: low SDI. SDI, socio-demographic index. ASIR, age-standardized incidence rate

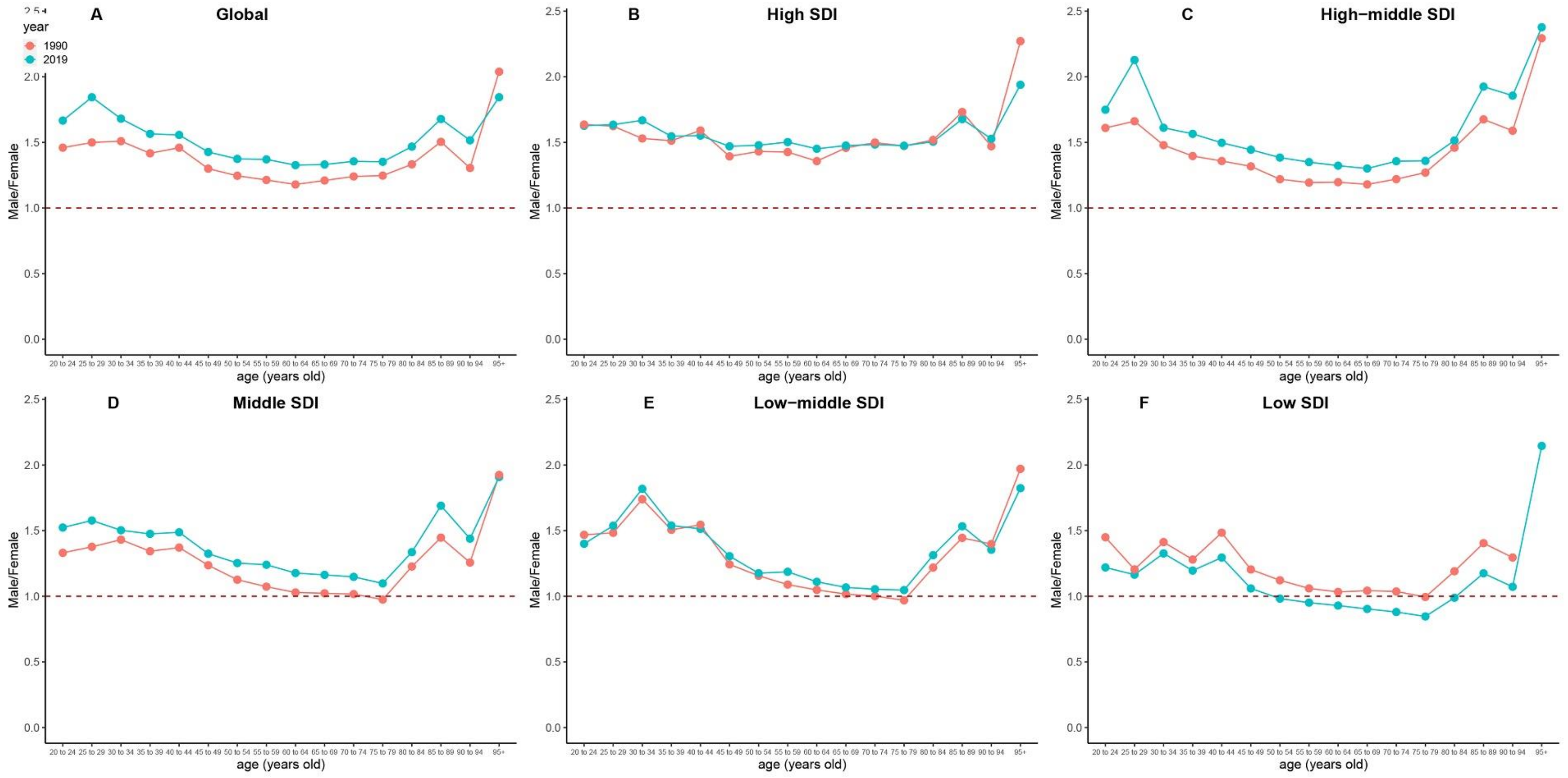

Supp Figure 5 Joinpoint regression analysis of age-standardized death rates (ASDR) among males and females from 1990 to 2019. APC: annual percent change. A: male; B: female. * Indicates that the APC is significant different from zero at the alpha = 0.05 level. ASDR, age-standardized death rate.

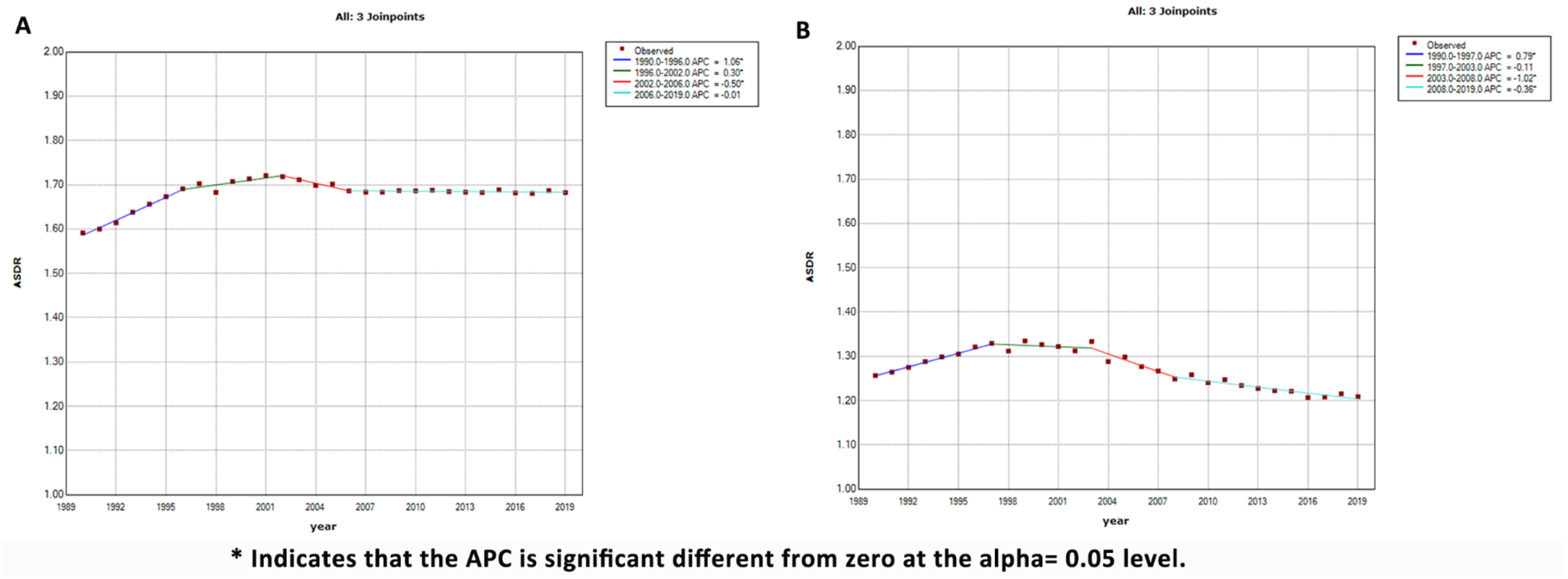

Supp Figure 6 The ASDR of multiple myeloma among regions based on SDI in 2019. ASDR: age standardized death rate (per 100,000 population).

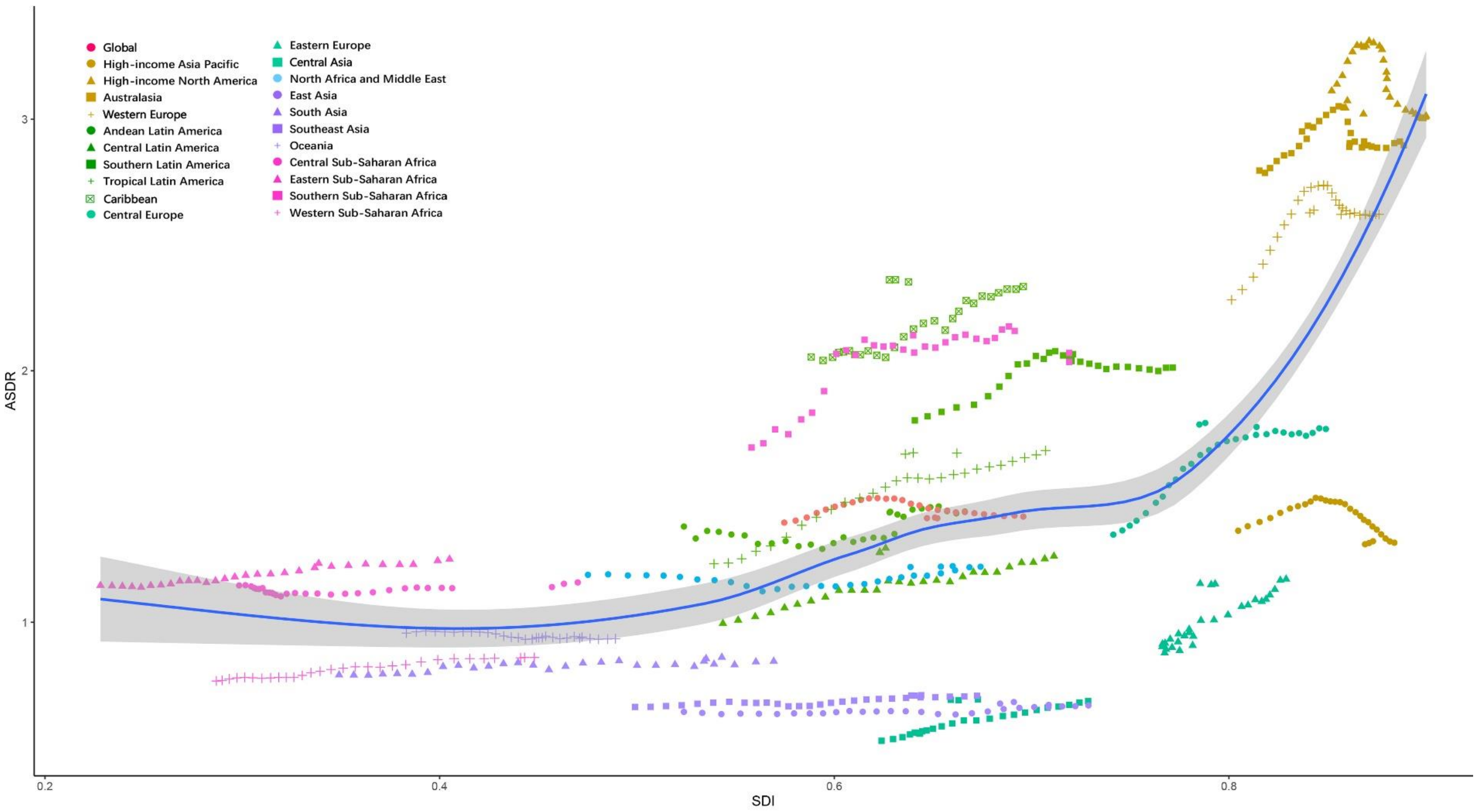

Supp Figure 7 T The ratio of male to female ASDR among different age groups in 1990 and 2019. A: global; B: high SDI; C: high-middle SDI; D: middle SDI; E: middle-low SDI; F: low SDI. SDI, socio-demographic index. ASDR, age-standardized death rate

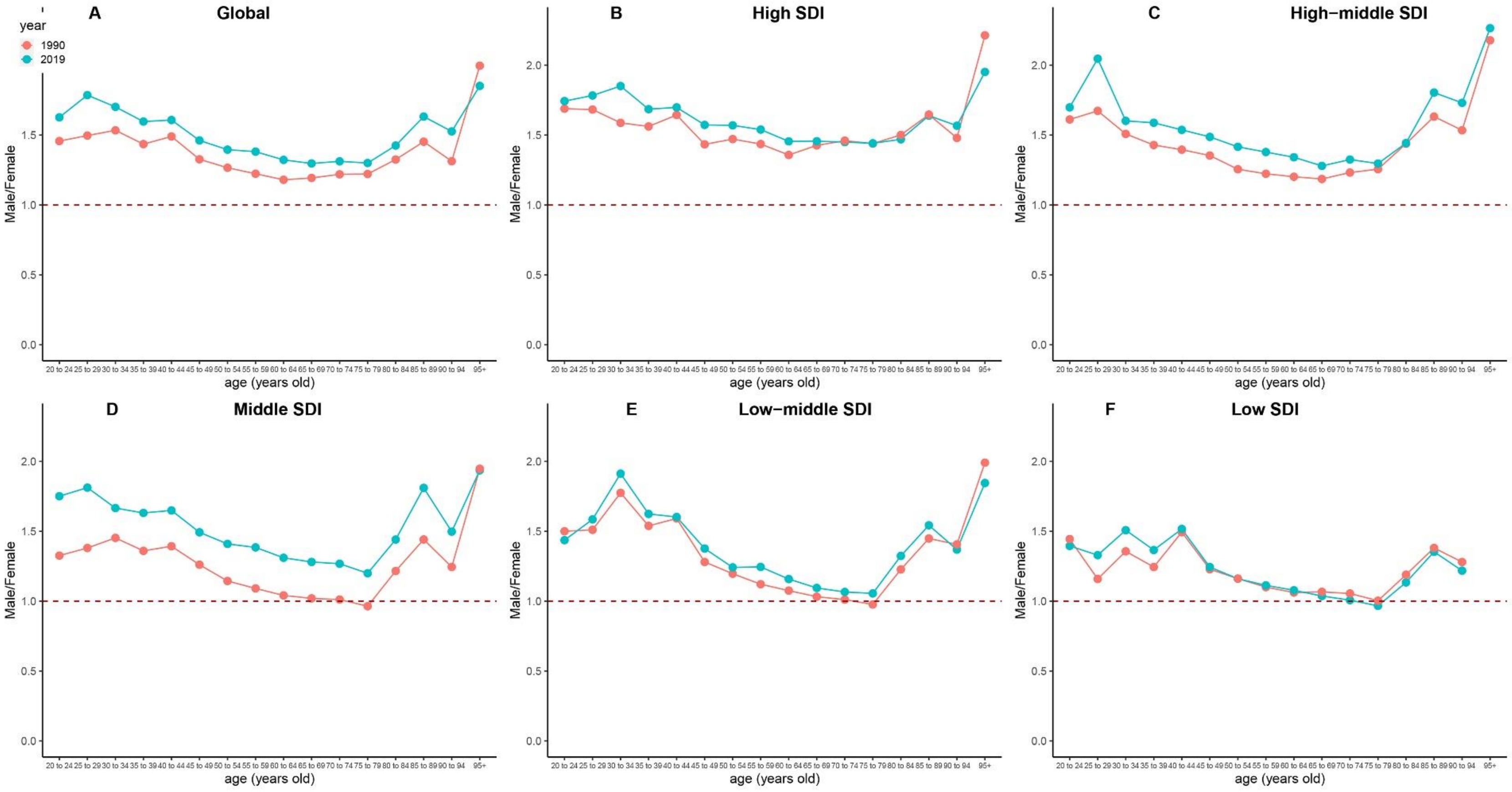

Supp Figure 8 The age-standardized DALYs rate of multiple myeloma among regions based on SDI in 2019.

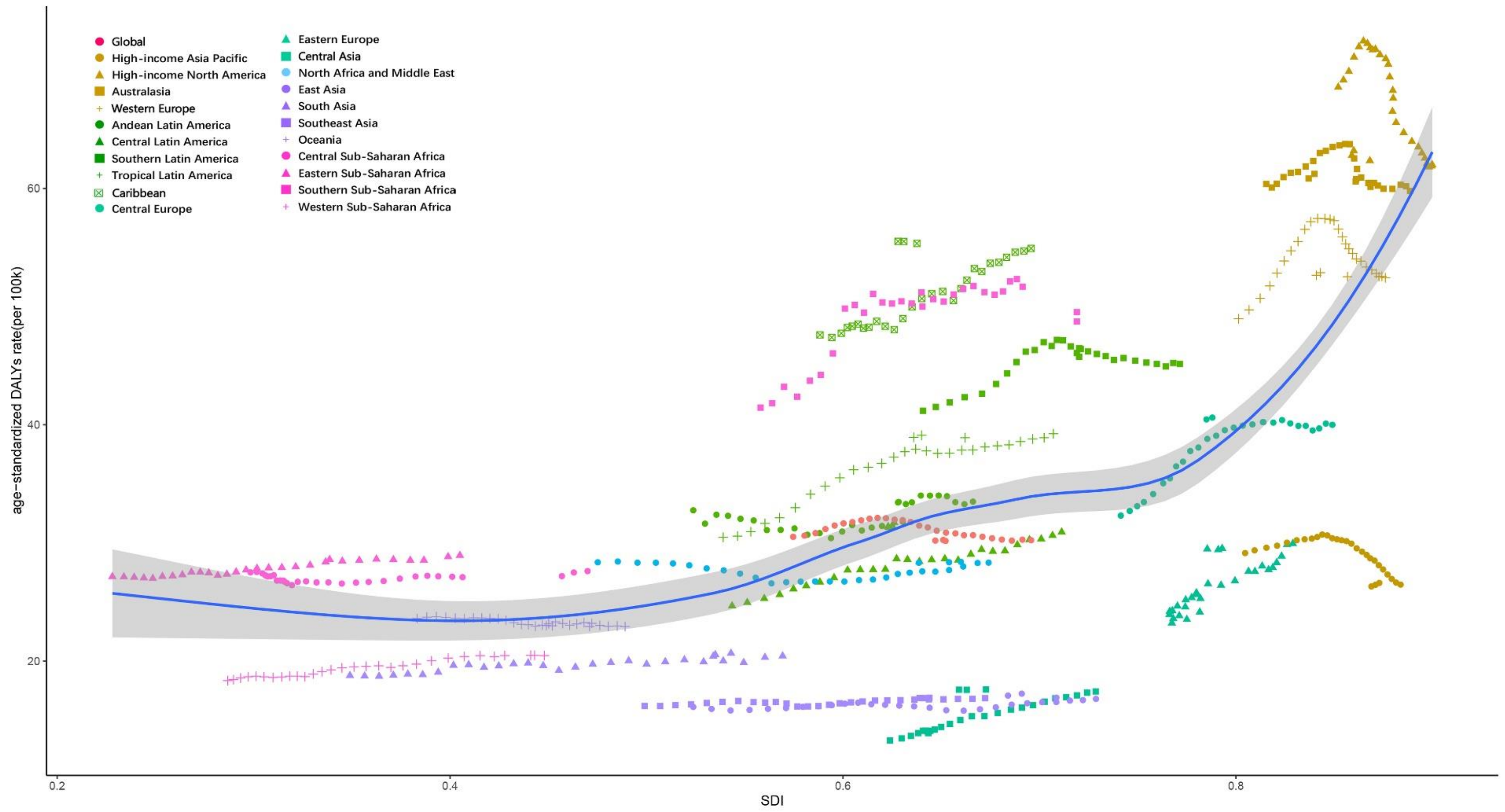

Supp Figure 9 The percentage of age-standardized deaths and DALYs attributable to high body-mass index from 1990 to 2019.

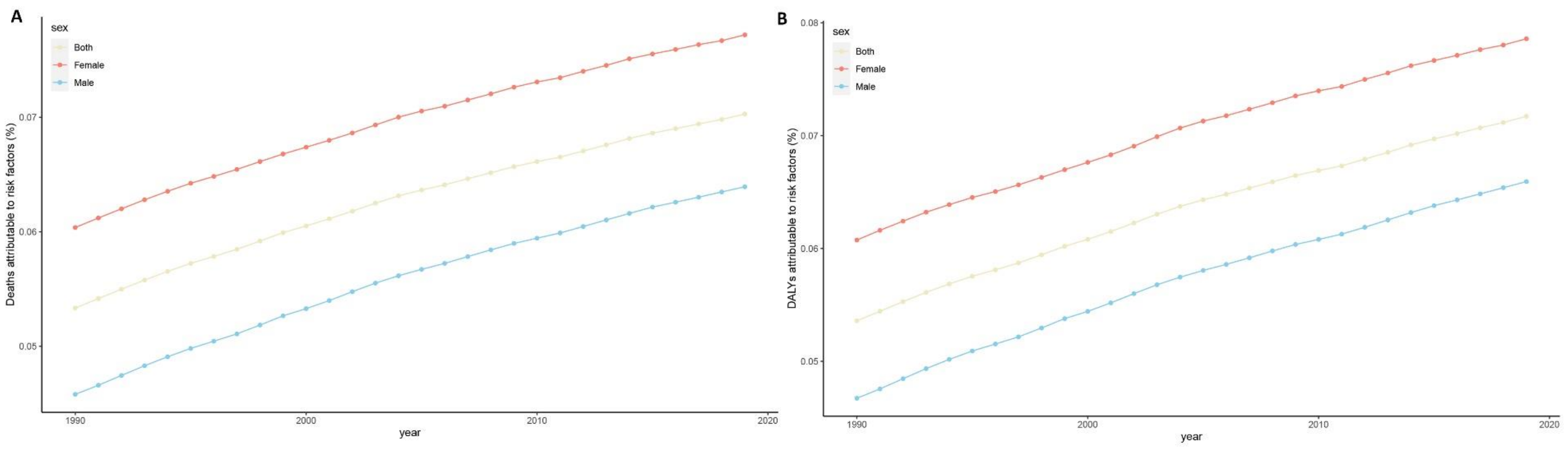

Correspondence with GBD Management Team (gbdsec@uw.edu)

RE: A request for use of GBD data

GBD Secretariat 发送给 zhoulinghui@zju.edu.cn

发起会议

2021-03-16 05:51:37

Dear Dr. Zhou,

Thank you for your email.

Data made available for download on IHME Websites can be used, shared, modified, or built upon by non-commercial users via the Open Data Commons Attribution License. This license is obtained by the institution so that the public may access data from use any information obtained as needed, provided that you cite the data. Learn more about the Open Data Commons Attribution License here. Our full Terms and Conditions can be found here.

We hope this is helpful to you and wish you the best of luck on your research. If you have further questions about the data, results or tools found on our website, please reach out any time.

Kind regards,
GBD Secretariat

———————— Original Message ————————
From: zhoulinghui@zju.edu.cn [zhoulinghui@zju.edu.cn]
Sent: 3/11/2021 8:29 PM
To: gbdsec@uw.edu
Subject: A request for use of GBD data

Dear GBD Collaborator Network:

I'm a doctor in Bone Marrow Transplantation Center, the First Affiliated Hospital, Zhejiang University School of Medicine. I'm interested in GBD study. So I intend to measure the global, regional, and national burden of multiple myeloma from 1990 to 2019 based on GBD data . Can you allow me to use the data on multiple myeloma.
I would appreciate it if you allow me to use the data.
These data are very important. The paper can not be completed without such data.
I am looking forward to hearing from you. Thank you very much.
Best,
Sincerely,

Linghui Zhou

zhoulinghui@zju.edu.cn

Institute for Health Metrics and Evaluation | University of Washington
Seattle, WA, USA | healthdata.org

ref:_00D4615j72._5004oJdGFe:ref
